# Simulation of the Impact of Pesticides on Pollinators Under Different Conditions Using Correlation Weighting of Quasi-SMILES Components Together with the Index of Ideality of Correlation (IIC)

**DOI:** 10.3390/jox16010010

**Published:** 2026-01-08

**Authors:** Alla P. Toropova, Andrey A. Toropov, Sofia Mescieri, Alessandra Roncaglioni, Emilio Benfenati

**Affiliations:** Department of Environmental Health Sciences, Istituto di Ricerche Farmacologiche Mario Negri IRCCS, Via Mario Negri 2, 20156 Milan, Italy; andrey.toropov@marionegri.it (A.A.T.); sofia.mescieri@marionegri.it (S.M.); alessandra.roncaglioni@marionegri.it (A.R.); emilio.benfenati@marionegri.it (E.B.)

**Keywords:** pollinators, CORAL software, Las Vegas algorithm, Monte Carlo method, pesticide toxicity, QSAR

## Abstract

*Background*: Pesticide toxicity to insects is an important adverse effect with a potentially large ecological impact when considering the effect on beneficial insects, as pollinators. The assessment of this endpoint is necessary to avoid applying ecologically dangerous pesticides. *Aim of the study:* Assessment of the availability of the Monte Carlo method for the development of a model for toxicity (pLD50) towards bees and other pollinators. In addition, the index of ideality of correlation is examined as a possibility to increase the statistical quality of quantitative structure–activity relationships (QSARs) for the toxicity of pesticides to pollinators. *Main results and novelty:* models with good performance on the toxic effect of pesticides towards different pollinators, wrapping acute and chronic effects, using the Monte Carlo method for QSAR analysis.

## 1. Introduction

Pollinators are essential components of terrestrial ecosystems and global agriculture, as a large proportion of crop species depend on insect-mediated pollination [1,2,3,4,5,6,7,8]. By ensuring the reproduction of wild and cultivated plants, they sustain biodiversity, ecosystem functioning and food security [2,4,5]. In Europe, 80% of the crops depend on pollinators, with an economic output of at least 5 billion euros [9]. Despite their ecological and economic importance, pollinators are increasingly exposed to multiple stressors, including habitat loss, pathogens, climate change and agrochemical use, among which pesticide exposure is recognized as a major driver of decline worldwide [8]. For these reasons, the protection of pollinators has become a growing priority in environmental risk assessment and the European Commission established an Action Plan to protect pollinators [9]. Honey bees are commonly used as sentinel organisms in studies of pesticide effects on pollinators [10,11], because they are widely distributed, relatively simple to monitor and have a concise and well-understood biological cycle [8,12,13]. However, the pollinator community is much broader, and other wild pollinators, such as *Bombus terrestris* spp., *Osmia* spp. and *Megachile rotundata*, play an equally crucial role in the maintenance of biodiversity, ecosystem functioning and agricultural productivity. Recent studies have emphasized the importance of extending pesticide toxicity assessments beyond honey bees to other species, in order to reduce existing data gaps and improve the ecological relevance of risk assessments [8,10,11,14].

Since experimental toxicity testing is costly and requires in vivo procedures, in silico approaches, such as quantitative structure–activity relationship (QSAR) models, have been recommended to estimate pesticide toxicity, and indeed QSAR models have been developed for honey bees, in particular addressing contact exposure [15,16,17,18,19,20,21,22,23]. The regression models [24,25,26,27] and models that include classification [28], distinguishing substances as toxic and non-toxic [29,30,31,32,33] using a threshold value, for instance, can predict the dose producing the adverse effect. The models used a range of molecular descriptors and algorithms. However, the studies on pollinators other than honey bees are very limited [20,33,34]. This scarcity of studies may be partly due to the fact that OECD test guidelines for bumblebees (adopted 2017) [35,36] and solitary bees such as Mason Bee (*Osmia* sp.) (adopted 2025) [37] are relatively recent and also to the limited availability of toxicity data for these pollinators compared to *Apis mellifera* [10,11,14]. Therefore, predictive models should not be limited to honey bees but should also include other pollinators to ensure a more effective pesticide risk assessment. The objective of this study is therefore to develop a QSAR model using toxicological data from different pollinator species by integrating experimental toxicity values from the databases with predicted environmental fate characteristics in order to improve ecological risk assessment. Furthermore, we addressed not only acute toxicity and effects towards adults and larvae, in order to have better tools to protect pollinators.

## 2. Materials and Methods

### 2.1. Data

A dataset containing 541 data points of chemicals tested on pollinators was compiled from the ECOTOX database [38] and the EFSA OpenFoodTox database [39]. Only studies reporting food intake (oral exposure) of active ingredients or mono-constituents with a purity of at least 80% were included. Preference was given to studies conducted following standardized OECD guidelines for oral acute [36,40] and chronic tests [41]: acute tests with exposure durations between 24 and 96 h and chronic tests of 10 days. In addition, subchronic tests of 5–8 days and chronic tests extending up to 14 days were also included to increase dataset coverage, particularly for non-Apis pollinators, for which experimental information is limited. All values were harmonized in µg/organism. The dataset includes acute and chronic oral toxicity values for adult and larval pollinators (*Apis mellifera*, *Apis mellifera linguistica*, *Apis mellifera carnica*, *Bombus terrestris*, *Bombus terrestris audax*, *Megachile rotundata*, *Osmia excavata*, *Osmia lignaria* and *Osmia rufa*), with exposure durations ranging from 1 to 14 days, expressed in µg/organism. Values indicated by the qualifiers “>” or “<” were used simply by considering their numerical value, in order to maximize the number of available data for non-Apis pollinators, where experimental data are generally scarce. Among these, 540 entries correspond to LD_50_ values and 1 to an LC_50_ value. We used canonical SMILES to verify the presence of duplicates. We removed a total of 159 duplicates, obtaining a dataset of 382 substances. The single LC_50_ data point in our dataset was not retained for model construction, because during the duplicate-removal step for the same molecule, a lower LD_50_ value was available.

Toxicity values (LD_50_ expressed in µg/organism) were converted into negative decimal logarithms (pLD_50_).

Physicochemical and environmental properties were predicted using VEGA (version 1.2.4) and JANUS (version 1.0.3) software, both available from VEGAHUB [42], including persistence in water, soil and sediment, expressed in days.

In this way, we obtained a dataset and generated quasi-SMILES for the substances [43,44]. The SMILES (Simplified Molecular-Input Line-Entry System) is a widely used format for processing chemical substances in QSAR. Quasi-SMILES contains the SMILES (preprocessed with VEGA, to obtain canonical, standardized SMILES) with additional labels, relative to the features as in Table 1. These features contain a label to specify the persistence of the substance in different compartments, the pollinator species, the life stage and the exposure duration.

**Figure 1:**
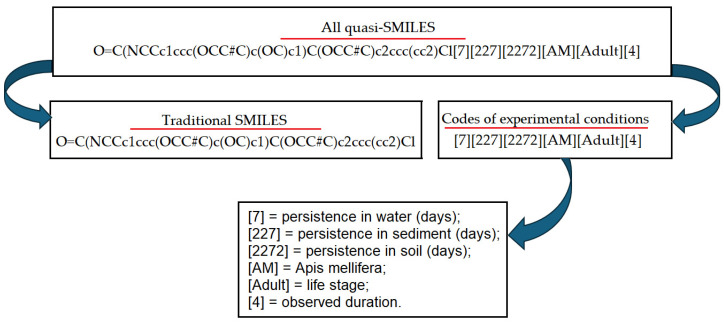
The scheme of quasi-SMILES construction. Underlines demonstrate the logic of quasi SMILES.

Figure 1 contains an example of quasi-SMILES construction. This is quite a clear action which is aimed at providing a simulation system by adding information about phenomena considered. In fact, the majority of phenomena in general and QSAR phenomena in particular are characterized by the Hilbert space of experimental (observed) conditions. In practice, it is impossible to separate the important and non-important conditions and circumstances. However, it is clear that the more circumstances involved in the simulation process, the higher the probability of obtaining more robust results.

But, on the other side, the large number of circumstances involved in the simulation process can lead to the extraordinary complexity of a model. In order to avoid this situation, it is necessary to limit the system at the basis of the simulation to a reasonable number of considered conditions. Table 1 contains the list of experimental and available considerations in the process of simulating the model for pesticide toxicity towards pollinators. Preliminary computational experiments have indicated that various kinds of persistence (in water, sediment and soil) are able to serve as a reliable basis for developing corresponding models for the toxicity of pesticides.

### 2.2. Simulation Scheme

The basic steps of building a model within the framework of the used method are as follows: (1) Splitting into active and passive training sets, calibration set, and validation set. (2) Optimizing the correlation weights of molecular features extracted from quasi-SMILES. (3) Building a regression model linking the descriptor calculated by the correlation weights with the endpoint under study (toxicity for bee and bumblebee). (4) Validating the predictive potential of the model.

Within step (1), the set of all compounds was randomly divided into four subsets of approximately the same number of quasi-SMILES: (i) an active training (≈25%), (ii) a passive training (≈25%), (iii) a calibration (≈25%) and (iv) a validation (≈25%). It should be noted that the active and passive training sets, together with the calibration set, are the training set in the traditional approach. However, in practical terms, the calibration set was used to generate the final, optimized model, while the active and passive training sets were used only in the initial steps, and thus, the models obtained with these subsets are preliminary ones. In step (2), the active training set was used to calculate the correlation weights, and then the passive training set was used as an inspector of the correlation weights supplied by the active training set. In step (3), the calibration set was used to check for overfitting. Finally, in step (4), the external validation set was used to evaluate the predictive potential of the resulting model. Since the validation set contains substances that are not visible in the calculation of the correlation weights, this step is useful to estimate the observed predictive potential. This process was repeated five times to obtain a reliable statistical basis, creating five random splits [45].

We clarify that, as described above, the set of quasi-SMILES has been randomly split into 4 subsets: active training set, passive training set, calibration set, and the validation set. None of the quasi-SMILES is present in more than one of these four subsets.

It should be noted that the approach under consideration, which involves considering multiple groups of splits for training and validation sets, requires a tool to verify that the splits under consideration are distinct. For this purpose, data on the identity of the splits under consideration is used. Table 2 presents the information, separated into the training and validation parts of the list, which is fully available for analyzing data on the used organic compounds. This means that the same quasi-SMILES can be present in both splits 1 and 2, for instance, but it cannot be present twice in the same split, as we wrote.

### 2.3. Optimal Descriptors

The optimal descriptors are the correlation weights for all non-rare quasi-SMILES attributes, summed within a single equation. The correlation weights are (i) attributes of traditional SMILES, and (ii) attributes indicated experimental conditions collected in Table 1. The non-rare quasi-SMILES are identified within the modelling development, as described below.

### 2.4. Optimization of Correlation Weights

The correlation weights of quasi-SMILES attributes are calculated using the CORAL software (version CORALSEA-2025) developed by Istituto Mario Negri, Milano, Italy [46]. The optimization process used here includes a special algorithm called Index of Ideality of Correlation (IIC) [47,48]. The algorithm avoids “local” situations, not valid from a general point of view. The initial modelling steps, as implemented using the active and passive sets, start from specific cases and can be tailored to the local situation. To obtain more general lessons, the prediction is improved using the calibration set, even if the statistics on the calibration set may be lower than those on the active and passive training sets (which may show higher values for overfitting). The overfitting can be controlled by using the weighting coefficients for the IIC. The choice of the coefficient is made empirically, based on the results of preliminary observations of the stochastic optimization system with different weights for the IIC.

Having the numerical data on the correlation weights, the considered toxicity to pollinators can be calculated using Equation (1):

(1) $$p{LD}_{50}=C_{0}+C_{1}\times DCW\left(T,N\right)$$

C_0_ and C_1_ are regression coefficients; T is the threshold used to define rare and non-rare SMILES attributes. The SMILES attribute is considered rare if its frequency in the active training set is less than T (rare attributes are not considered; their correlation weights are equal to zero). N is the number of epochs of Monte Carlo optimization. In this study, T = 3 and N = 15 were obtained from computational experiments within the modelling optimization, as in Equation (2).

(2) $$DCW\left(T,N\right)=\sum {CW(S}_{k})+\sum CW({SS}_{k})$$

For the calculation of the correlation weights necessary for calculating the descriptors by Equation (1), the following target functions were used, and, in particular, IIC was used in Equation (4):

(3) $${TF}_{0}=R_{AT}+R_{PT}-\left|R_{AT}-R_{PT}\right|\times 0.1$$

(4) $${TF}_{1}={TF}_{0}+IIC\times 0.3$$

R_AT_ and R_PT_ are correlation coefficients between observed and calculated values of pLD_50_ observed for the active training and passive training sets, respectively.

A clear advantage of the Monte Carlo method for constructing the models is its ability to build a prediction based on a simple representation of the molecular structure by SMILES, without the need to take into account their spatial structure and complex descriptors based on representations of atom–atom potentials and quantum mechanical interpretations of charges and electronic density distributions.

**Figure 2:**
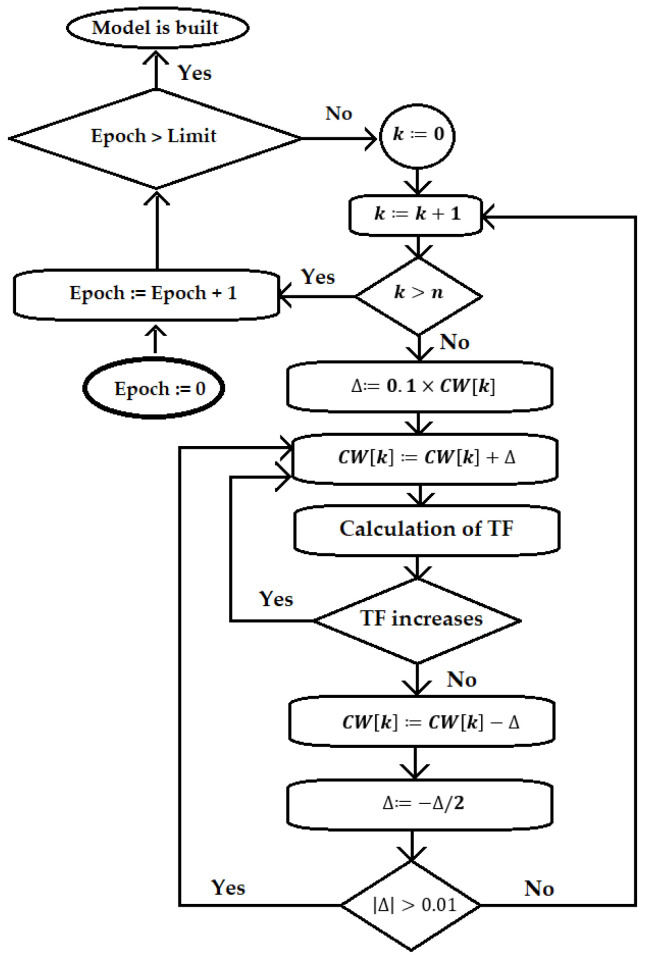
The block scheme of the Monte Carlo optimization.

Figure 2 shows the scheme of the optimization used in this study.

**Figure 3:**
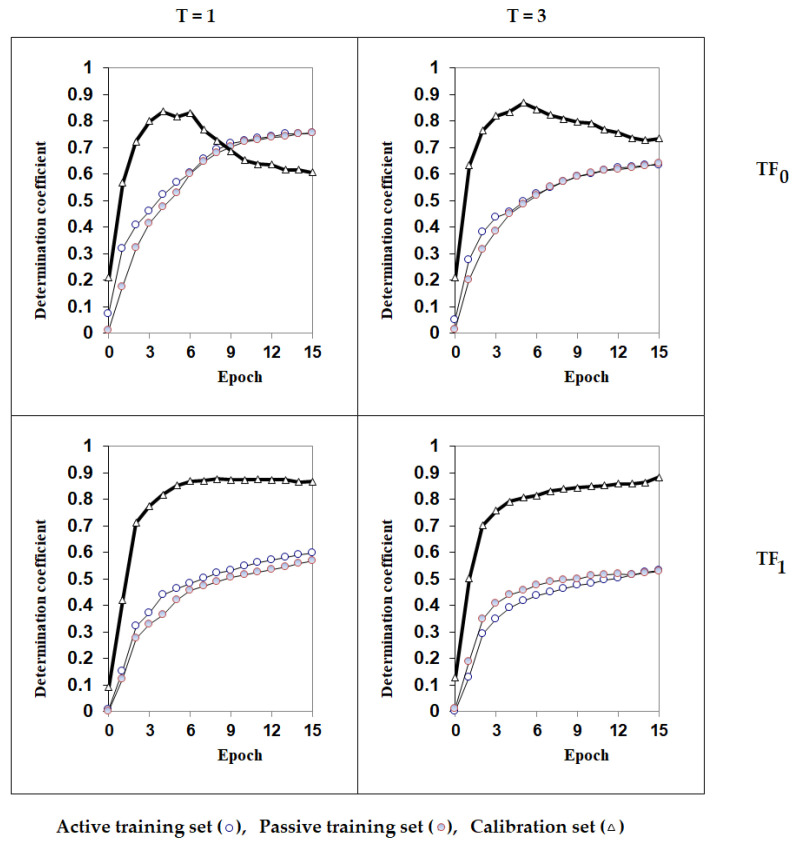
The results of the modelling process performed within the progressive epochs of the Monte Carlo optimization with different thresholds (T) and target functions.

Figure 3 shows the sequence of the process of the Monte Carlo optimization. One can see that the selection of the threshold 3 (used in Equation (2)) gives better statistical results for the calibration set for both TF0- and TF1-optimization. In the case of TF0, the number of epochs of the optimization selected is equal to six (Figure 3). In the case of TF1, the results slightly improved with the number of epochs, and we stopped the model at 15 epochs. The Monte Carlo process is a statistical procedure, and thus, results may vary. The statistical parameters shown in Figure 3 exhibit clear dispersion, and there may be different ideal values across several runs of the optimization under the same split in active, passive training, calibration, and validation sets. We have empirically selected reasonable T and N values, also to limit the time needed for the process, observing that the modifications on the different epochs were minimal with a high number of epochs.

### 2.5. Applicability Domain

The applicability domain for the described model, calculated with Equation (1), defines the so-called statistical defects of SMILES attributes. These defects can be calculated as follows:

(5) $$d_{k}=\frac{\left|{P(A}_{k})-{P\prime (A}_{k})\right|}{N\left(A_{k}\right)+N\prime (A_{k})}+\frac{\left|{P(A}_{k})-{P\prime \prime (A}_{k})\right|}{N\left(A_{k}\right)+N\prime \prime (A_{k})}+\frac{\left|{P\prime (A}_{k})-{P\prime \prime (A}_{k})\right|}{N\prime \left(A_{k}\right)+N\prime \prime (A_{k})}$$

where P(A_k_), P′(A_k_) and P″(A_k_) are the probability of A_k_ in the active training set, passive training set, and calibration set, respectively; N(A_k_), N′(A_k_) and N″(A_k_) are frequencies of A_k_ in the active training set, passive training set, and calibration set, respectively. The statistical SMILES-defects (D_j_) are calculated as

(6) $$D_{j}=\sum_{k=1}^{NA}d_{k}$$

where NA is the number of non-blocked SMILES attributes in the SMILES.

A SMILES falls in the domain of applicability if

(7) $$D_{J}<2\times \bar{D}$$

The results of the process related to the applicability domain are reported in Table S1 in Supplementary Materials, showing the outliers on split-1. The results presented in Section 3 consider all substances, without excluding outliers. The outliers for splits 1, 2, 3, 4 and 5, respectively, are 11, 14, 12, 16 and 13.

### 2.6. Mechanistic Interpretation

Several runs of the CORAL program can provide the basis for a mechanistic interpretation of quasi-SMILES codes. Using numerical data on the correlation weights of features obtained in several Monte Carlo optimization runs; two types of these features can be distinguished: (1) features with a positive correlation weight for all program runs. Such features can be considered as promoters of the growth of the values of the endpoint under study. (2) Features with only a negative correlation weight in all runs. These features can be classified as promoters of a decrease in the value of the endpoint under study. All other features cannot be considered as promoters of an increase or decrease for the endpoint under study.

## 3. Results

QSAR models have been obtained with the CORAL software [46], using quasi-SMILES [43,44] that represent in the same format the information on the substance, and the result of the experiment about toxicity towards different pollinators with different exposure durations; further data on the environmental persistence are codified within the quasi-SMILES too. The equation has been optimized using two target functions, TF_0_ and TF_1_. Table 3 contains the results for the training and validation set for five different splits in the case of the TF_0_ and TF_1_. One can see that in the case of TF_1,_ the predictive potential of the models is better in comparison with the case using TF_0_. The results are presented for the five splits to evaluate if there is consistency among splits and thus sound results. The results are shown for the different sets of substances. The active and passive training sets are those used in the initial phases of the modelling process. In these phases, the final model is not yet developed. Indeed, the model is optimized using the calibration set. Thus, the statistical values related to the final model are those indicated for the calibration model. The validation set contains substances not used for the model development. Table 3 provides the results of the different statistical evaluation checks that we did. All these parameters show that the models using TF_1_ are better. Let us consider the values of the determination coefficient D obtained with the TF_0_. They are summarized in Table 4.

**Figure 4:**
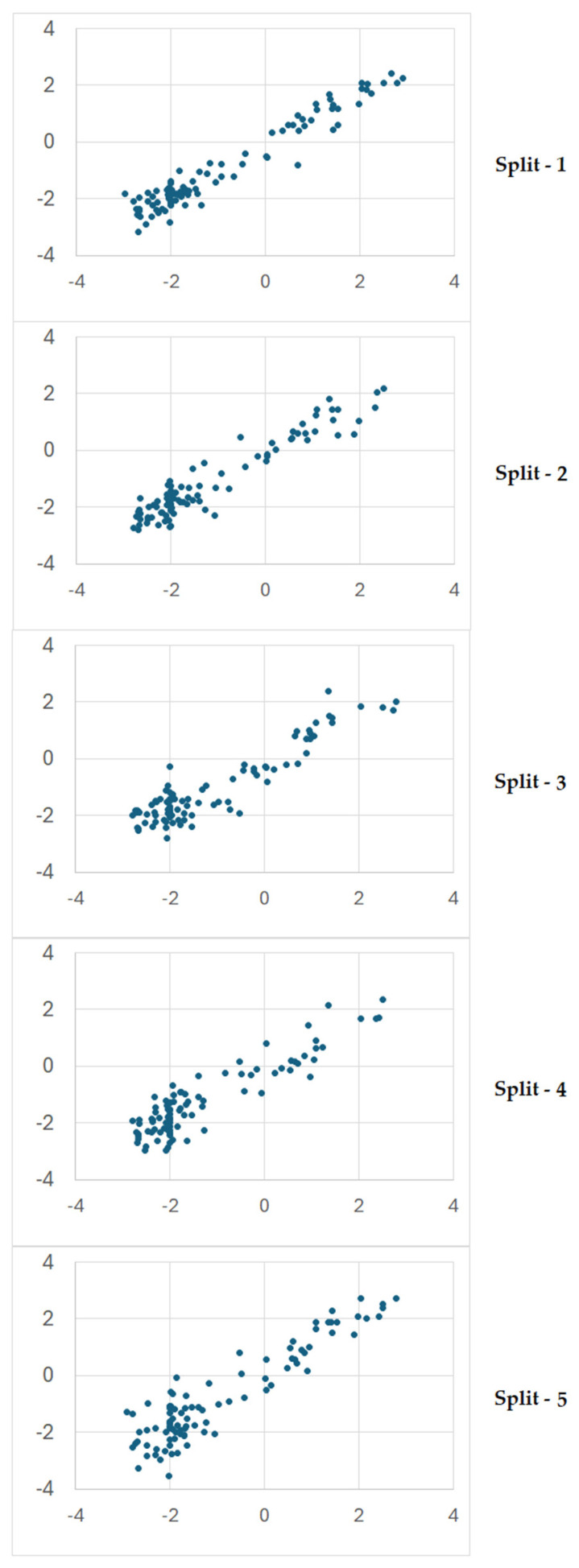
Plots of experimental vs. calculated values of the endpoint for splits 1–5. On the *x*-axis, there are the experimental values. On the *y*-axis, there are the predicted values. Dots represent the toxicity values.

Figure 4 contains the plots of experimental vs. calculated values of the endpoint for the five splits.

Table 4 shows the average values of the determination coefficient for the calibration and validation sets for the TF_0_ and TF_1_.

Table 4 represents the results on the calibration set, which are those of the final model developed. The validation set contains substances not used to develop the model. Thus, the results on the validation set provide an indication of the expected results when the model is used for new substances. Table 4 clearly shows the improvement obtained using TF_1_, which uses IIC too. There is an improvement of 0.11 for both the calibration and validation sets. The results of the validation and calibration sets are very similar, indicating the model is predictive also towards new substances and is quite robust. In addition to the improvement of the average value, Table 4 shows that the range of the values obtained replicating the models using the five different splits is much smaller in the case of the TF_1_, which indicates that the models are more stable and reproducible.

Although the model was developed using a pooled dataset, each data point is explicitly associated with a specific pollinator species, life stage (adult or larva) and exposure duration. To assess the ecological and regulatory relevance of the model, we evaluated its predictive performance across the most representative data subsets. In particular, model performance was analyzed separately for major taxonomic groups (*Apis* spp., *Bombus terrestris* spp. and *Osmia* spp.), life stages (adults and larvae) and exposure durations (acute, subchronic and chronic). The results of this subgroup analysis for the calibration and validation sets are summarized in Table 5.

As shown in Table 5, the model exhibited consistently high predictive performance across the most represented pollinator taxa. Performance for *Apis* spp., which constitutes the largest subset, was strong (R^2^ = 0.91, RMSE = 0.46), supporting the robustness of the model for honey bees. Despite the smaller number of data points, the model also performed well for *Bombus terrestris* spp. (R^2^ = 0.97, RMSE = 0.25) and *Osmia* spp. (R^2^ = 0.92, RMSE = 0.36), suggesting good generalization.

When stratified by life stage, predictive performance was slightly higher for adults (R^2^ = 0.94) than for larvae (R^2^ = 0.84), likely reflecting greater biological variability and higher uncertainty in larval toxicity data. Performance statistics could not be computed for *Megachile rotundata*, as no data points for this species were included in the calibration or validation sets.

Regarding exposure duration, the model showed high performance for acute exposures (R^2^ = 0.93), while performance decreased for subchronic exposures (R^2^ = 0.70), consistent with the smaller size and higher heterogeneity of this subset. The chronic subset contained too few data points to allow robust conclusions, although the reported metrics are provided for completeness.

Table S1 in the Supplementary Material contains the results of the model observed for split 1 using TF_1_ optimization, together with data on numerical values of the descriptor as well as the quasi-SMILES configurations.

## 4. Discussion

The new approach applied within this study is to address simultaneously multiple pollinators, with data across different exposure and life stages. To achieve this, on a methodological point of view, we applied (1) the quasi-SMILES technology and (2) the index of ideality of correlation. The quasi-SMILES, as shown in Table 1 and Figure 1 in this specific case, contains information on the chemical substance through the SMILES structure. The use of SMILES for QSAR is very common. What is new here is that the quasi-SMILES includes in the code additional pieces of information. In this case, information on the different species of pollinators is present, extending the models not only to honey bees. Furthermore, the duration of exposure is taken into account within the quasi-SMILES string. Finally, the environmental behaviour of the substance, in particular persistence in water, soil and sediment, is used in the quasi-SMILES. In this way, the model largely extends its applicability, covering a range of situations, and exploits environmental data that may play a role in the overall impact of the pesticides. In this way, the model applies to multiple species and covers multiple exposure durations. The fixed exposure duration, used to define the different kinds of protocols for toxicity assessment towards bees, is useful to gather standardized data in a reproducible way. Conversely, the adverse effects of the same substance in different pollinators may be observed at different times, and thus the model presented here is effective in covering effects appearing at different times depending on the substance and species. This approach is aligned with the framework of the Dynamic Energy Budget (DEB), which explores the complexity of the phenomena leading to toxic effects occurring in different taxa and at different times [50]. From a methodological point of view, the approach we present is convenient because it allows us to exploit data that are sparse and poorly represented in some circumstances.

The development of multiple individual models specific to each species and at different times would be impossible due to the lack of sufficient data suitable to represent all cases. Conversely, merging all data and organizing it into a coherent scheme, as in the present case, allows extending the modelling approach to situations that take advantage of associated experimental observations on related substances or species or durations. We clarify that the lack of data is always an issue, and this approach cannot cover all situations. We expect that there is less uncertainty for predictions related to honey bees, which is more broadly represented in the training set, compared with other species, and the same applies for the evaluation at different durations. Further data are needed to better verify the performance and the different pollinators for chronic exposure.

Another limitation regards the interpretation related to the biological relevance and the differences among species. Further information related to the traits of the different pollinators may be used for this purpose, when available.

Regarding the algorithm, without quasi-SMILES or without supplementing traditional SMILES with codes of experimental condition, the set of objects under consideration exhibits a high degree of degeneracy (identical, indistinguishable traditional SMILES). Without the use of the IIC, the statistical quality of the forecast is significantly lower (Table 3 and Table 4). The IIC implements an algorithm able to focus the attention of the model towards substances representing the “correct” correlation between experimental and calculated values, and vice versa, able to “avoid” signals derived from uncommon substances, which will be outliers. There are many studies in the direction of algorithms for attention, also in other areas, such as generative artificial intelligence. Our algorithm proved to be effective in prioritizing the most relevant parts of the quasi-SMILES. Thus, the above ideas can be considered as useful tools for the QSAR analysis of toxicity towards pollinators. It is to be noted that the basic idea of the approach considered is the Monte Carlo method.

As shown in Table 5, the subgroup analysis supports the applicability of the quasi-SMILES-based approach across different pollinator taxa, life stages and exposure durations, while also revealing clear data limitations for non-Apis species and longer exposure durations that constrain the statistical evaluation of model performance.

Table 6 contains attributes of quasi-SMILES that are promoters of an increase or decrease in pesticide toxicity in pollinator insects. One can see that the presence of branching in the molecular structure (denoted by brackets), carbon in the sp^2^ state, double bonds, chlorine and nitrogen are promoters of toxicity increase. This is explainable by the fact that, for instance, branching of the carbon structure is often associated with higher hydrophobicity. The carbon in the sp^2^ state and double bonds are often associated with higher reactivity of the molecule. Chlorinated pesticides are often more toxic than others. Nitrogen, and in particular substances with nitro groups, are often more toxic than others. Conversely, the presence of cycles (denoted by digits), pairs of connected carbon atoms in the sp^3^ state, as well as the presence of sulfur atoms, are promoters of toxicity decrease. This series of attributes has a statistical meaning and it may help in the identification of potential mechanisms. We clarify that the proposed assumptions regarding the decrease and increase in toxicity towards pollinators are justified on a probabilistic point of view, because they have been identified by the software considering the population of substances in the training and validation sets. It is useful to make reasoning about the possible mechanisms and associations, as exemplified above. However, the overall process codified by the software is more complex, and it cannot be simplified with a few linear relationships with individual features. Given that most natural phenomena are complex, it is appropriate to address them in a probabilistic way. At the same time, this property of this method conceals a drawback: this approach lacks robust universality and the ability to standardize, because it is strictly associated with the specific set of substances and features used. New substances and new experimental conditions may modify the population of items and conditions, with the possibility of further improvements.

Table 7 contains a comparison of the models for bee acute toxicity (oral exposure) suggested in the literature.

One can see that the statistical quality of the models suggested here is better. The advantages are in the highest number of substances (reflected also in the higher number of substances in the validation set), in the higher value of the determination coefficients, and in the lower value of the root mean square error. In addition, this model works not only on acute toxicity and not only on honey bees, thus these two are other advantages, not represented in Table 7. There are other advantages, compared with the model published by Moreira-Filho et al. [29]: that model relies on feed-forward neural networks (FNN) and requires molecular descriptors (and fingerprints for classification), making it dependent on feature generation and preprocessing. In contrast, the quasi-SMILES CORAL regression developed here requires only SMILES strings and no descriptor or fingerprint calculation, while directly encoding species identity, exposure duration and physicochemical persistence within the same quasi-SMILES representation. Overall, the present approach offers a simpler, descriptor-free and computationally lighter alternative to existing neural network models, while expanding prediction capability beyond *Apis mellifera* and beyond a single oral toxicity endpoint.

It is worth noting that only a limited number of studies have developed regression-based QSAR models for acute oral endpoints. As we wrote, there are more studies on contact exposure. If we compare the results we obtained with those from published QSAR models on contact toxicity (thus through a different exposure route) [19,31,32], the set of substances used in the other studies is much lower (in the range of 6 to 17 substances), with determination coefficients in the range of 0.84–0.96. Thus, the performance of our model is quite similar to or better than what was achieved in other studies on contact exposure, but the advantages that we mentioned above remain. For instance, Hamadache et al. [19] used 1666 molecular descriptors starting from the 3D molecular structure and then used neural networks. This requires much more effort to achieve the 3D structure, introduces uncertainty regarding the 3D optimization, needs preprocessing of the molecules and complex molecular descriptors, and sophisticated algorithms. Some models are focused on specific chemical classes. In another study, Dulin et al. only addressed carbamates and organophosphorus substances [31]. They used 3D structures, 2489 molecular descriptors, and Genetic Function Approximation (GFA), producing 500 equations. Thus, also in this case, it is clear that a major effort is needed to generate models. We developed models for honey bees too [25,26,30,32]. In some cases, we used a previous version of CORAL and fewer substances [25,30]. In another study, a complex approach was performed using hybrid models, thus developing two models based on molecular descriptors, which were then combined into a single model [32].

Overall, the current approach provides one of the few available oral toxicity QSARs in pollinators, while being simpler to implement, trained on a richer dataset, applicable to multiple species (*Apis mellifera* spp., *Bombus terrestris* spp., *Osmia* spp. and *Megachile rotundata*), two different life stages (adult and larva), and exposure durations (acute, subchronic, and chronic), and capable of delivering predictive performance comparable or superior to existing models. By requiring only SMILES as input and avoiding complex descriptor calculations, this framework represents a robust and versatile predictive tool for pollinator risk assessment.

Last, but not least, the approach described has been applied in different QSPR/QSAR studies in the aspect of using the quasi-SMILES methodology [51,52,53,54,55,56,57,58,59,60,61,62,63,64] as well for the investigation of the IIC as a tool to improve the predictive potential [65,66,67,68,69,70,71,72,73,74,75,76,77,78,79,80,81,82,83].

## 5. Conclusions

We introduced a general model approach to the toxicity of pesticides towards pollinators, not focused on honey bees. Results were good for different pollinators, exposure time and life stage. This is relevant to introduce tools useful for the protection of wild pollinators, accounting for their important ecological and agricultural role. The novel approach also goes beyond the exposure times with a fixed granularity, which may fail to capture the effects occurring at times different from the official ones. The quasi-SMILES technique gives models of pesticide toxicity to pollinators of quite satisfactory quality. IIC improved the predictive potential of models. Heuristically, the approach proposed here represents a convenient palette of possibilities for solving similar problems related to QSAR analysis. In purely computational terms, the above-mentioned correlation ideality index represents an opportunity to examine previously unapplied correlation quality. Our study provides recommendations for effectively managing the stochastic selection process of correlation weights, which have a significant influence on the mechanistic interpretation. Due to its statistical basis, the uncertainty of the model is lower for conditions that are better represented, such as honey bees and acute exposure; further data may increase the confidence (or possibly allow the development of better models in the future) on less represented conditions, such as chronic toxicity or *Osmia* species.

## Figures and Tables

**Table 1:** Features of quasi-SMILES used for modelling.

CODE	COMMENT
WT	Persistence in water [days]
SS	Persistence in sediment [days]
SO	Persistence in soil [days]
SP	Species
LS	Life stage
OB	Observation duration [days]

**Table 2:** Percentage of identity for the five studied splits into the training and validation set.

	1	2	3	4	5
**1**	100	39.4 *	34.4	44.8	36.1
**2**	40.2	100	42.9	38.7	37.3
**3**	32.5	36.5	100	34.7	45.8
**4**	33.5	44.8	41.2	100	38.5
**5**	45.3	42.9	35.2	37.3	100

* If i > j, then the matrix element [i,j] means the percentage of identity for the active training sets; if i < j, then the matrix element [i,j] means the percentage of identity for the validation sets (external sets). The i (i.e., for the columns) and j (for the rows) mean the numbering of the 5 splits examined.

**Table 3:** The statistical characteristics of models observed for five splits into the training (active-, passive-, and calibration) and validation sets.

Target Function	Split	Set *	n	D	CCC	IIC	Q^2^	<R_m_^2^>	MAE	F	Na
TF_0_	1	A	97	0.6157	0.7621	0.7376	0.5987		0.848	152	
		P	94	0.6157	0.7732	0.7058	0.6000		0.728	147	
		C	97	0.8028	0.8949	0.7521	0.7946	0.7564	0.552	387	
		V	94	0.8570	-	-	-	-	0.51	-	100
	2	A	96	0.6629	0.7973	0.8142	0.6502		0.770	185	
		P	96	0.6659	0.7941	0.7818	0.6501		0.777	187	
		C	95	0.7192	0.8471	0.8106	0.7096	0.6700	0.655	238	
		V	95	0.7725	-	-	-	-	0.56	-	96
	3	A	95	0.6607	0.7957	0.6172	0.6451		0.762	181	
		P	96	0.6548	0.7969	0.7190	0.6415		0.720	178	
		C	94	0.8728	0.9226	0.6259	0.8677	0.8498	0.569	631	
		V	97	0.8096	-	-	-	-	0.61	-	101
	4	A	95	0.6993	0.8230	0.7526	0.6887		0.698	216	
		P	95	0.7285	0.8134	0.5765	0.7169		0.846	249	
		C	95	0.6479	0.7966	0.7356	0.6350	0.5732	0.815	171	
		V	97	0.6871	-	-	-	-	0.77	-	99
	5	A	97	0.7316	0.8450	0.8040	0.7180		0.609	259	
		P	94	0.7313	0.8487	0.7829	0.7196		0.662	250	
		C	95	0.7106	0.8343	0.7874	0.6977	0.6425	0.731	228	
		V	96	0.6644	-	-	-	-	0.77	-	100
TF_1_	1	A	97	0.4855	0.6537	0.4890	0.4603		1.01	90	
		P	94	0.4798	0.6635	0.5363	0.4566		0.932	85	
		C	97	0.9102	0.9491	0.9537	0.9069	0.8380	0.383	963	
		V	94	0.9405	-	-	-	-	0.32	-	100
	2	A	96	0.5303	0.6930	0.5908	0.5127		0.957	106	
		P	96	0.5274	0.7047	0.5737	0.5042		0.959	105	
		C	95	0.8832	0.9344	0.9394	0.8792	0.8589	0.416	703	
		V	95	0.9025	-	-	-	-	0.35	-	96
	3	A	95	0.5090	0.6746	0.4756	0.4842		0.949	96	
		P	96	0.4869	0.6608	0.4876	0.4665		0.981	89	
		C	94	0.8452	0.9064	0.9193	0.8392	0.7715	0.509	502	
		V	97	0.8335	-	-	-	-	0.46	-	101
	4	A	95	0.5211	0.6851	0.6228	0.5015		0.975	101	
		P	95	0.6771	0.7500	0.4740	0.6627		0.920	195	
		C	95	0.8356	0.9102	0.9139	0.8294	0.7929	0.487	473	
		V	97	0.8177	-	-	-	-	0.48	-	99
	5	A	97	0.5875	0.7402	0.6363	0.5670		0.864	135	
		P	94	0.5875	0.7530	0.7039	0.5690		0.903	131	
		C	95	0.8492	0.9171	0.9182	0.8423	0.7722	0.488	524	
		V	96	0.8507	-	-	-	-	0.50	-	100

* A = active training set; P = passive training set; C = calibration set; and V = validation set; *n* = the number of quasi-SMILES in a set; D = determination coefficient; CCC = concordance correlation coefficient; IIC = index of ideality of correlation; Q^2^ = cross validated correlation coefficient (should be >0.6); <R_m_^2^> = Roy and Kar metric [49] (should be >0.6); MAE = mean absolute error; F = Fischer F-ration; Na = the number of active quasi-SMILES attributes.

**Table 4:** The average values of the determination coefficient for the calibration and validation sets for the TF_0_ and TF_1_ (in parentheses, the range of the values in the five splits).

	TF_0_	TF_1_
Calibration set	0.75 (0.65–0.87)	0.86 (0.84–0.91)
Validation set	0.76 (0.66–0.86)	0.87 (0.82–0.94)

**Table 5:** Model performance by species, life stage and exposure duration on calibration and validation sets.

Subgroup	N	R^2^	RMSE
*Apis* spp.	175	0.91	0.46
*Bombus terrestris* spp.	9	0.97	0.25
*Megachile rotundata*	-	-	-
*Osmia* spp.	7	0.92	0.36
Adults	123	0.94	0.43
Larvae	68	0.84	0.47
Acute	143	0.93	0.44
Subchronic	45	0.70	0.46
Chronic	2	0.78	0.23

**Table 6:** List of promoters for the increase and decrease in pesticide toxicity. Promoters of toxicity decrease are indicated by grey.

Attribute of Quasi-SMILES	CWs * Run 1	CWs Run 2	CWs Run 3	CWs Run 4	CWs Run 5	NA	NP	NC	S_k_
C...(.......	0.0779	0.2255	0.1014	0.0347	0.1229	91	89	93	0.0002
c...c.......	0.0766	0.0026	0.0844	0.3675	0.2334	78	68	77	0.0007
Cl..(.......	0.1406	0.2713	0.2941	0.2074	0.4830	58	55	51	0.0009
N...(.......	0.1919	0.0678	0.2758	0.1823	0.2387	56	58	61	0.0006
=...(.......	0.5996	0.6721	0.4614	0.1333	0.5304	55	52	59	0.0007
n...c.......	0.3686	0.1722	0.3637	0.2684	0.6192	46	40	45	0.0007
N...C.......	0.7570	0.9766	0.9795	0.6388	0.7433	42	38	43	0.0006
c...C.......	1.4670	1.9743	1.8103	1.3863	2.0857	32	29	28	0.0009
c...O.......	0.2966	0.3994	0.6405	0.1038	0.4649	24	16	20	0.0026
1...........	−0.1586	−0.3426	−0.3408	−0.3667	−0.1228	95	83	89	0.0007
1...(.......	−0.2410	−0.4791	−0.4058	−0.2481	−0.1908	49	38	42	0.0016
C...C.......	−0.3833	−0.1924	−0.2086	−0.2670	−0.3992	45	47	49	0.0006
S...(.......	−0.1742	−0.2165	−0.1575	−0.2682	−0.4748	14	22	23	0.0031

* CW = correlation weights. The attributes associated with a decrease in toxicity are in a grey background. NA, NP and NC are frequencies of the quasi-SMILES code in active training, passive training and calibration sets, respectively.

**Table 7:** Comparison of the results in validation of QSAR models of pollinators’ toxicity (oral exposure).

Number of Compounds in Validation Set	Determination Coefficient	Root Mean Squared Error	Reference
28	0.75	0.68	[29]
94	0.94	0.43	Best model in this work

## Data Availability

The original contributions presented in this study are included in the article/Supplementary Material. Further inquiries can be directed to the corresponding author.

## Supplementary Materials

The following supporting information can be downloaded at: https://www.mdpi.com/article/10.3390/jox16010010/s1, Table S1: Technical details for split 1.
